# Molecular insights into Adgra2/Gpr124 and Reck intracellular trafficking

**DOI:** 10.1242/bio.021287

**Published:** 2016-12-09

**Authors:** Naguissa Bostaille, Anne Gauquier, Laure Twyffels, Benoit Vanhollebeke

**Affiliations:** 1 Laboratory of Neurovascular Signaling, Department of Molecular Biology, ULB Neuroscience Institute, Université libre de Bruxelles (ULB), Gosselies B-6041, Belgium; 2 Center for Microscopy and Molecular Imaging, Université libre de Bruxelles (ULB), Gosselies B-6041, Belgium

**Keywords:** Adgra2/Gpr124, Reck, Wnt7, Zebrafish, Leucine-rich repeat, Blood-brain barrier

## Abstract

Adgra2, formerly known as Gpr124, is a key regulator of cerebrovascular development in vertebrates. Together with the GPI-anchored glycoprotein Reck, this adhesion GPCR (aGPCR) stimulates Wnt7-dependent Wnt/β-catenin signaling to promote brain vascular invasion in an endothelial cell-autonomous manner. Adgra2 and Reck have been proposed to assemble a receptor complex at the plasma membrane, but the molecular modalities of their functional synergy remain to be investigated. In particular, as typically found in aGPCRs, the ectodomain of Adgra2 is rich in protein-protein interaction motifs whose contributions to receptor function are unknown. In opposition to the severe *ADGRA2* genetic lesions found in previously generated zebrafish and mouse models, the zebrafish *ouchless* allele encodes an aberrantly-spliced and inactive receptor lacking a single leucine-rich repeat (LRR) unit within its N-terminus. By characterizing this allele we uncover that, in contrast to all other extracellular domains, the precise composition of the LRR domain determines proper receptor trafficking to the plasma membrane. Using CRISPR/Cas9 engineered cells, we further show that Adgra2 trafficking occurs in a Reck-independent manner and that, similarly, Reck reaches the plasma membrane irrespective of Adgra2 expression or localization, suggesting that the partners meet at the plasma membrane after independent intracellular trafficking events.

## Abstract (precis)

**Summary:** This work uncovers molecular determinants of Adgra2/Gpr124 and Reck trafficking to the plasma membrane where the partners meet to act as potent Wnt7-specific Wnt/β-catenin signaling co-activators required for brain vascularization.

## INTRODUCTION

Adhesion G protein-coupled receptors (aGPCRs) constitute the second largest group of GPCRs in humans. Most aGPCRs are orphan receptors with no identified ligands that function through remarkably diverse mechanisms (Fredriksson et al., 2003; Hamann et al., 2015). They differ from other GPCRs by long N-terminal extensions preceding a membrane-proximal GPCR autoproteolysis-inducing (GAIN) domain containing the highly conserved GPCR proteolytic site (GPS) (Araç et al., 2012). These N-terminal sequences typically comprise multiple protein-protein interaction domains involved in cell-cell and cell-matrix contacts. This structural hallmark significantly broadens the signaling potential and complexity of this class of GPCRs that, context-dependently, behave as adhesion molecules or signal transducing GPCRs (Hamann et al., 2015). ADGRA2, a member of this branch of GPCRs previously known as GPR124, has gained considerable interest since the discovery of its essential role in brain vascular development (Kuhnert et al., 2010). Upon genetic inactivation, vascularization and blood-brain barrier maturation are impaired in all or parts of the zebrafish and mouse central nervous system, respectively (Anderson et al., 2011; Cullen et al., 2011; Kuhnert et al., 2010; Vanhollebeke et al., 2015). This receptor promotes angiogenic sprouting through endothelial cell (EC)-autonomous Wnt/β-catenin signaling stimulation upon contact with neural progenitor-derived Wnt7 ligands (Posokhova et al., 2015; Vanhollebeke et al., 2015; Zhou and Nathans, 2014).

Genetic studies in zebrafish have shown that in order to recognize these ligands, and hence to be competent for brain invasion, ECs must additionally express Reck, a GPI-anchored glycoprotein (Ulrich et al., 2016; Vanhollebeke et al., 2015). Consistently, EC-specific invalidation of *RECK* in the mouse leads to CNS-specific vascular defects, thereby demonstrating the evolutionary conserved role of *RECK* in cerebrovascular development (de Almeida et al., 2015). Adgra2 and Reck have been proposed to interact at the plasma membrane to assemble a potent and Wnt7-specific Wnt/β-catenin co-activator complex (Vanhollebeke et al., 2015). The complex also operates in neural crest-derived cells to promote dorsal root ganglia (DRG) neurogenesis in zebrafish embryos (Prendergast et al., 2012; Vanhollebeke et al., 2015). Defective DRG neurogenesis is accompanied by metamorphic pigmentation alterations in the adult *adgra2* mutant skin (Vanhollebeke et al., 2015).

While the genetic interaction between *adgra2* and *reck* is well supported by studies in the zebrafish model as well as cell culture experiments, their activation and signaling mechanisms are poorly characterized (Noda et al., 2016; Vanhollebeke et al., 2015). We therefore need to better define the cellular and molecular modalities of the Adgra2/Reck synergistic interaction. In particular, the stoichiometry of the Adgra2/Reck complex and the molecular determinants of its trafficking, assembly and signal transduction still need to be investigated. The N-terminal domains of Adgra2 are likely contributors to several, if not all, of these processes. Indeed, cell culture and *in vivo* experiments have revealed that Adgra2 function critically relies on its extracellular domain architecture. N-terminal truncations or substitution of the ectodomain of Adgra2 with the equivalent domain derived from the closely related Adgra3, abrogate receptor signaling (Posokhova et al., 2015; Vanhollebeke et al., 2015). Moreover, the Adgra2 potential interaction interface with Reck, a cell surface exposed GPI-anchored glycoprotein, is restricted to the extracellular parts of the receptor.

**Fig. 1.**
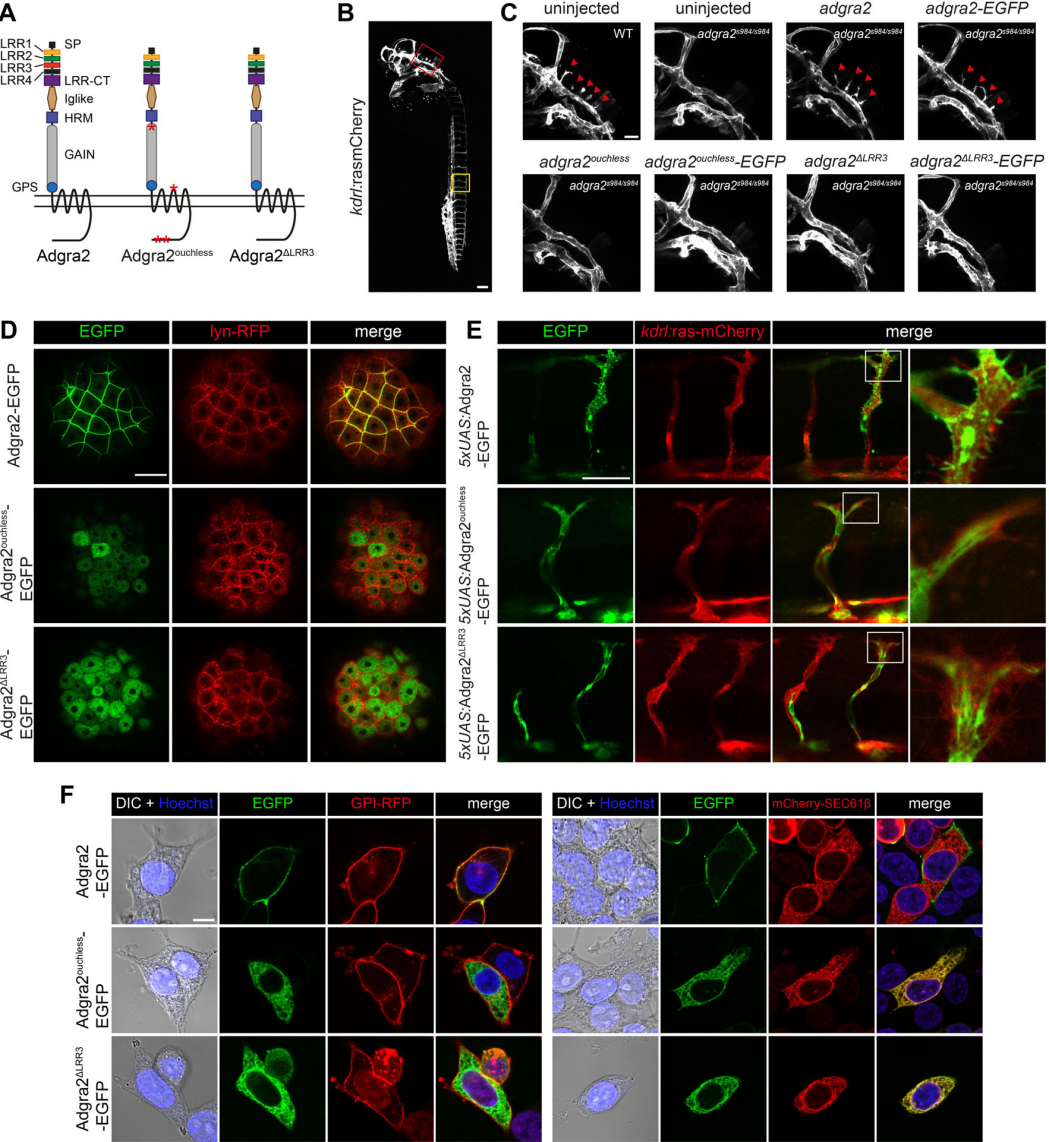
**Adgra2^ouchless^ mislocalizes to the endoplasmic reticulum.** (A) Schematic representation of Adgra2, Adgra2^ouchless^ and Adgra2^ΔLRR3^ topology and domain organization. Adgra2^ouchless^ and Adgra2^ΔLRR3^ lack the third LRR motif (red rectangle). The positions of the residue variations resulting from naturally occurring SNPs in *adgra2^ouchless^* are designated by red asterisks. (B) Maximal intensity projection of a confocal z-stack of a WT *Tg(kdrl:ras-mCherry)* embryo at 36 hpf in lateral view. The red and yellow boxes define, respectively, the magnified areas of the hindbrain vasculature shown in C and the intersegmental vessels shown in E. Scale bar: 100 µm. (C) Maximal intensity projection of a confocal z-stack of WT and *adgra2^s984/984^ Tg(kdrl:ras-mCherry)* embryos at 36 hpf in lateral view after injection of 100 pg of *adgra2*, *adgra2-EGFP*, *adgra2^ouchless^*, *adgra2^ouchless^-EGFP*, *adgra2^ΔLRR3^* or *adgra2^ΔLRR3^-EGFP* mRNA at the one-cell stage. The red arrowheads point to the CtAs invading the hindbrain rhombomeres. Scale bar: 50 µm. (D) Single-plane confocal scans through enveloping layer cells of 5 hpf blastulas injected at the one-cell stage with 50 pg of *lyn-RFP* mRNA together with 100 pg of *adgra2-EGFP*, *adgra2^ouchless^-EGFP* or *adgra2^ΔLRR3^-EGFP* mRNA. Scale bar: 50 µm. (E) Single-plane confocal scans through the trunk intersegmental vessels of 30 hpf double-transgenic *Tg(kdrl:ras-mCherry); Tg(fliep:Gal4FF)* embryos injected at the one-cell stage with 25 pg of Tol2 transposase mRNA and 25 pg of the pTol2-*5xUAS:adgra2-EGFP*, pTol2-*5xUAS:adgra2^ouchless^-EGFP* and pTol2-*5xUAS:adgra2^ΔLRR3^-EGFP* constructs. Boxes define magnified views of the tip cells presented in the column on the right. Scale bar: 50 µm. (F) Single-plane direct fluorescence confocal scans of non-permeabilized HEK293T cells 48 h after transfection with GPI-RFP, mCherry-SEC61β, Adgra2-EGFP, Adgra2^ouchless^-EGFP or Adgra2^ΔLRR3^-EGFP encoding constructs. Cells were additionally transfected with *reck* and *Wnt7a* (mouse gene) expression constructs. Nuclei were counterstained with Hoechst. Scale bar: 10 μm.

As is typically found in aGPCRs, the extracellular N-terminus of Adgra2 comprises multiple protein-protein interaction domains whose contributions to receptor function remain largely elusive (Hamann et al., 2015). Specifically, the Adgra2 ectodomain is sequentially composed of an N-terminal LRR/CT domain, an Ig-like domain and a hormone receptor motif (HRM) preceding the membrane-proximal GPS-containing GAIN domain (Araç et al., 2012) (Fig. 1A). The Adgra2 LRR/CT domain contains four leucine-rich repeat (LRR) units which are 20-29 residue-long structural units that assemble in a superhelical manner with tandemly arranged repeats to form curved solenoid structures acting as protein interaction frameworks (Kobe and Kajava, 2001). As found in Adgra2, extracellular LRR motifs are often flanked by cysteine-rich C-terminal domains (LRR-CTs) that are integral parts of the LRR domain and shield the hydrophobic core of the last LRR motif. In this work, we will refer to the entire domain as LRR/CT and to the subdomain composed of the four LRR motifs as LRR.

Building a proper understanding of Adgra2 function will benefit from delineating the contribution of each N-terminal domain to receptor function. An Adgra2 variant exhibiting an altered N-terminal domain architecture was recently identified in the zebrafish *ouchless* mutants (Bostaille et al., 2017). As the result of an ENU-induced essential splice site mutation, the *ouchless* allele encodes an inactive and alternatively spliced *adgra2* (*adgra2^ouchless^*) lacking the third LRR motif of the LRR/CT domain. The zebrafish *ouchless* mutant thereby constitutes the first *in vivo* model of *adgra2* N-terminal domain-specific variation.

In this work, starting from the observation that the Adgra2^ouchless^ variant mislocalizes to the endoplasmic reticulum (ER), we undertook a comparative analysis of the contribution of the different Adgra2 N-terminal domains to Adgra2 and Reck intracellular trafficking and function. Detailed mutagenesis and chimeragenesis reveals that the LRR/CT domain controls Adgra2 trafficking. Investigations in genetically-engineered cultured cells further suggest that Adgra2 and Reck proceed independently through the secretory pathway and hence tentatively assign their synergistic effect on Wnt7-stimulated Wnt/β-catenin signaling to subsequent events occurring at the level of the plasma membrane.

## RESULTS

### Adgra2^ouchless^ accumulates within the endoplasmic reticulum

The *adgra2* variant found in *ouchless* mutants differs from *adgra2* reference sequences by four non-synonymous SNPs as well as a 72 bp deletion corresponding to exon 4 (Fig. 1A). While the SNPs represent naturally occurring variations, the exon 4 skipping event is caused by an ENU-induced essential splice-site mutation at the exon 4–intron 4 boundary and was shown to result in Adgra2^ouchless^ inactivation (Bostaille et al., 2017). Exon 4 encodes the third LRR motif (LRR3) of the LRR/CT domain. In order to determine how the absence of LRR3 mechanistically impairs Adgra2 function, we generated C-terminal EGFP-tagged versions of wild-type (WT) Adgra2 as well as *ouchless* (Adgra2^ouchless^) and ΔLRR3 (Adgra2^ΔLRR3^) variants. This latter variant reproduces the exon 4 deletion found in *ouchless* in a WT allele of *adgra2*, and hence lacks the *ouchless*-associated SNPs (Bostaille et al., 2017). We first evaluated the functionality of the fusion proteins in brain angiogenic assays in zebrafish by mRNA injections at the one-cell stage. While ectopic restoration of either EGFP-tagged or untagged versions of WT Adgra2 could restore angiogenic sprouting in *adgra2^s984/s984^* hindbrains (red arrowheads in Fig. 1C), the equivalent Adgra2^ouchless^ and Adgra2^ΔLRR3^ variants were inactive (Fig. 1B,C). These observations extend and confirm previous findings indicating that C-terminal fusions are compatible with receptor function *in vivo* and that, in the absence of LRR3, Adgra2 is non-functional (Vanhollebeke et al., 2015, Bostaille et al., 2017).

**Fig. 2.**
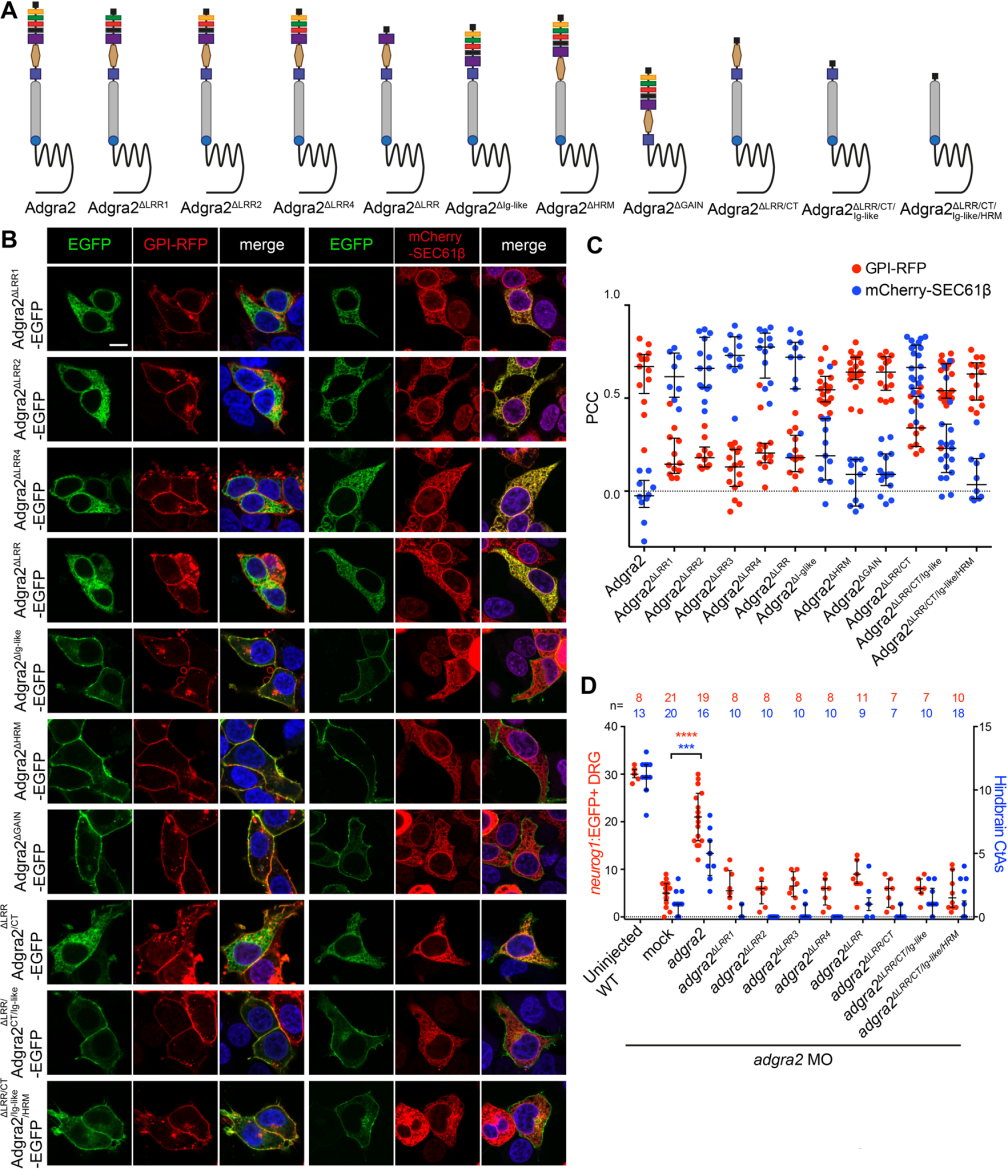
**LRR/CT-dependent Adgra2 intracellular trafficking.** (A) Schematic representation of Adgra2, Adgra2^ΔLRR1^, Adgra2^ΔLRR2^, Adgra2^ΔLRR4^, Adgra2^ΔLRR^, Adgra2^ΔIg-like^, Adgra2^ΔHRM^, Adgra2^ΔGAIN^, Adgra2^ΔLRR/CT^, Adgra2^ΔLRR/CT/Ig-like^ and Adgra2^ΔLRR/CT/Ig-Like/HRM^ domain organization. See Fig. 1A for schematic labels. (B) Single-plane direct fluorescence confocal scans of non-permeabilized HEK293T cells 48 h after transfection with the indicated *adgra2* variants together with the *GPI-RFP* membrane marker or the *mCherry-SEC61β* ER marker. Cells were additionally transfected with *reck* and *Wnt7a* (mouse gene) expression constructs. Nuclei were counterstained with Hoechst. Scale bar: 10 μm. (C) Colocalization assessment of Adgra2 and its variants with the membrane marker GPI-RFP (red dots) or the ER marker mCherry-SEC61β (blue dots) using the Pearson correlation coefficient. Error bars represent median±interquartile range. (D) Quantification of *neurog1:*EGFP^+^ DRG at 72 hpf (red dots) and hindbrain CtAs at 60 hpf (blue dots) in WT and *adgra2* morphant larvae and embryos injected at the one-cell stage with 100 pg RNA encoding Adgra2 or Adgra2 variants. Error bars represent median±interquartile range (***P<0.001; ****P<0.0001; Kruskal–Wallis test).

We then analyzed the stability and subcellular distribution of the EGFP-tagged variants in different cell types. When examined in the large and cobblestone-shaped enveloping layer cells of the 5 h post fertilization (hpf) zebrafish blastula, WT Adgra2-EGFP labeled the plasma membrane where it colocalized with a membrane-tethered lyn-RFP marker (Fig. 1D). By contrast, the mutant fusion proteins accumulated in an intracellular reticulate compartment reminiscent of the ER (Fig. 1D). Similarly, when analyzed in ECs of mosaic transgenic zebrafish, the WT fusion decorated the EC plasma membranes, including the numerous filopodial extensions of the tip cells, while the mutant variants showed strong intracellular and perinuclear signals that did not colocalize with the ras-mCherry EC membrane marker (Fig. 1E). Finally, in order to streamline quantitative colocalization studies, we imaged the cellular distribution of the EGFP fusion proteins in cultured HEK293T cells (Fig. 1F). Whereas the WT fusion protein accumulated at the plasma membrane marked by GPI-RFP as anticipated, the mutant versions failed to reach this compartment but instead accumulated intracellularly. The accumulating compartment was identified as the ER with the help of the mCherry-fused ER protein translocation apparatus component SEC61β (Fig. 1F). This was further quantitatively evaluated by Pearson's colocalization coefficient (PCC) analysis (Fig. 2C, see also Materials and methods). In all evaluated cell types, the intensity of the EGFP signals was comparable between WT and mutant Adgra2 fusions, indicating that the mislocalization does not trigger overt protein degradation under the experimental conditions used in these analyses.

### The LRR/CT domain controls Adgra2 trafficking

The mislocalization of the ΔLRR3 variant prompted us to perform a more detailed molecular dissection of the impact of the LRR/CT domain on Adgra2 progression through the secretory pathway. In-frame deletion of any of the four LRR repeats individually (ΔLRR1-4) or together (ΔLRR) resulted in ER retention (Figs 1 and 2A-C). This is a unique attribute of the LRR domain, as variants lacking one of the other domains individually (ΔIg-like, ΔHRM, ΔGAIN) reached the plasma membrane alike to WT Adgra2 (Fig. 2A-C).

Mechanistically, the LRR domain could be directly involved in trafficking through its recognition by an ER-resident binding partner that would assist Adgra2 progression. Alternatively, the absence or alteration of the LRR domain could act indirectly, for example by affecting Adgra2 folding. In the first scenario, the LRR domain should be strictly necessary for trafficking and hence any receptor deletion variant encompassing the LRR domain is predicted to accumulate in the ER. This was tested by analyzing the intracellular distribution of increasingly larger deletion variants with deletions ranging from the first LRR motif to the LRR C-terminal domain (ΔLRR/CT), the Ig-like domain (ΔLRR/CT/Ig-like) or the HRM domain (ΔLRR/CT/Ig-like/HRM). While the ΔLRR/CT variant exhibited an intermediate phenotype, with the most protein within the ER and a minor pool at the plasma membrane (Fig. 2A-C), the more severe deletion variants reached the plasma membrane akin to WT Adgra2. This latter observation is best explained by an indirect role of LRR/CT on Adgra2 trafficking, as discussed below. When assessed in zebrafish after mRNA injections at the one-cell stage, the ER-retained LRR/CT deletion variants did not exhibit angiogenic or neurogenic activity (Fig. 2D). However, we note that despite their correct localization at the plasma membrane, the multi-domain deletion variants were equally inactive suggesting that the LRR/CT domain or the adjacent Ig-like domain also contribute to later aspects of Adgra2 function, possibly related to Reck binding or Wnt7 recognition.

### Specific LRR3 amino acids govern Adgra2 trafficking

**Fig. 3.**
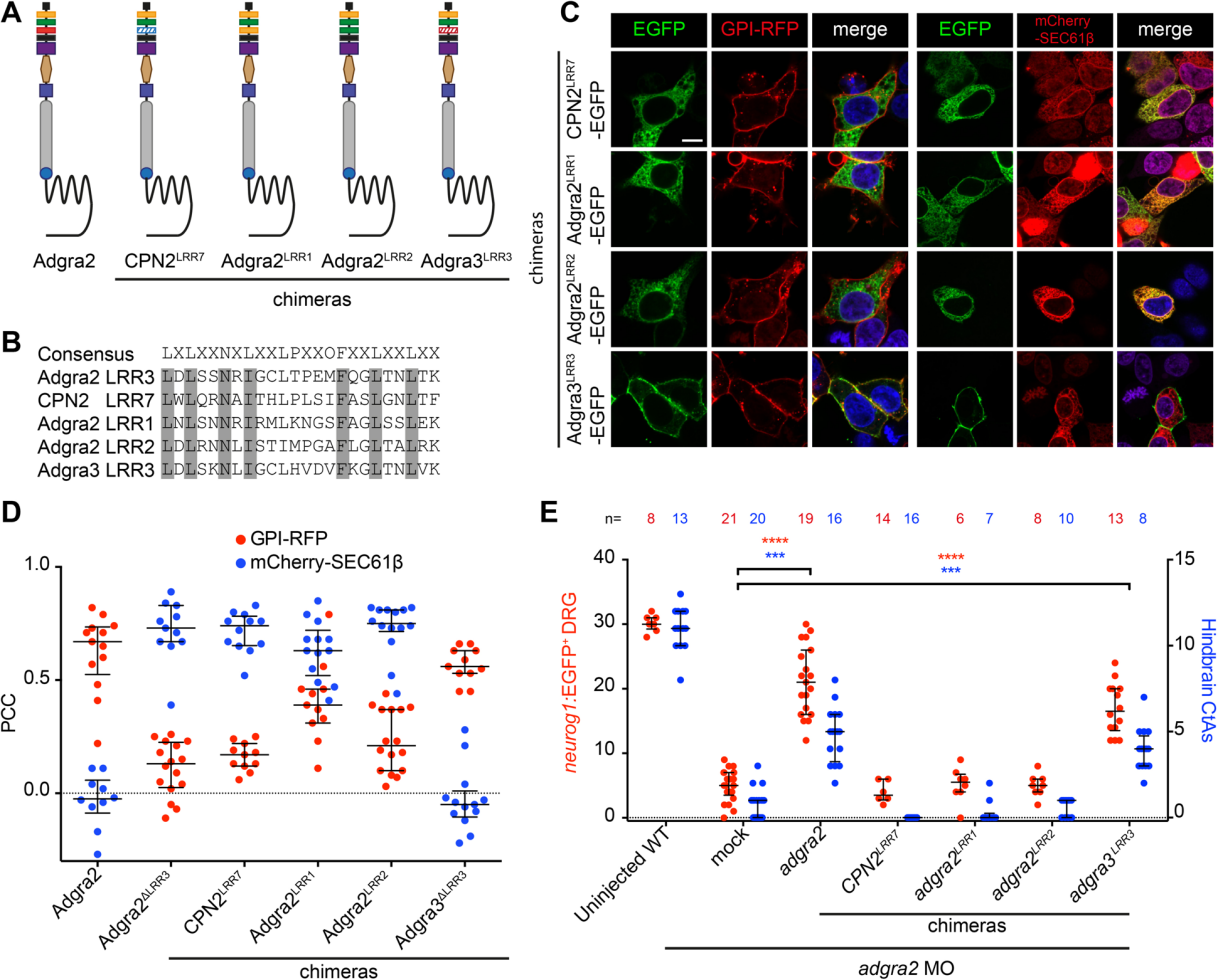
**Cellular distribution of Adgra2 LRR/CT domain hybrids.** (A) Schematic representation of chimeric Adgra2 receptors in which Adgra2 LRR3 is substituted with the LRR7 (blue hashed rectangle) of CPN2 (CPN2^LRR7^), the LRR1 of Adgra2 (Adgra2^LRR1^), the LRR2 of Adgra2 (Adgra2^LRR2^) or the LRR3 (red hashed rectangle) of Adgra3 (Adgra3^LRR3^). See Fig. 1A for schematic labels. (B) Sequence alignment of the LRR motifs illustrated in A. Identical amino acids are highlighted in gray. (C) Single-plane direct fluorescence confocal scans of non-permeabilized HEK293T cells 48 h after transfection with GPI-RFP, mCherry-SEC61β and Adgra2 hybrid-encoding constructs. Cells were additionally transfected with *reck* and *Wnt7a* (mouse gene) expression constructs. Nuclei were counterstained with Hoechst. Scale bar: 10 μm. (D) Colocalization assessment of Adgra2 and its variants with the membrane marker GPI-RFP (red dots) or the ER marker mCherry-SEC61β (blue dots) using the Pearson correlation coefficient. Error bars represent median±interquartile range. (E) Quantification of *neurog1:*EGFP^+^ DRG at 72 hpf (red dots) and hindbrain CtAs at 60 hpf (blue dots) in WT and *adgra2* morphant larvae and embryos injected at the one-cell stage with 100 pg of *adgra2* or *adgra2* hybrid mRNA. Error bars represent median±interquartile range (***P<0.001; ****P<0.0001; Kruskal–Wallis test).

LRR domains are composed of tandemly-arranged units that organize in arched solenoid assemblies contributing to the overall three-dimensional arrangement of proteins. Deleting one unit is thus anticipated to impact on the spatial arrangement of adjacent protein domains. The essential trafficking role revealed by the LRR/CT deletion variants could thus reflect a mere structural role of this domain that would fulfill its function sequence-independently. In agreement with this hypothesis, it has been previously demonstrated that the LRR/CT domain of Adgra2 can be substituted with the equivalent domain of Adgra3, a closely related but distinct aGPCR (Posokhova et al., 2015). We extended this analysis by generating chimeric receptors in which Adgra2 LRR3 is replaced by LRR motifs of different origins (Fig. 3A,B). When tested in HEK293T cells, the mislocalization in the ER was still observed upon substitution of Adgra2 LRR3 with LRR7 of human carboxypeptidase N subunit 2 (CPN2), LRR1 of Adgra2 or LRR2 of Adgra2. By contrast, LRR3 from zebrafish Adgra3 (Li et al., 2013) appears to be functionally interchangeable with Adgra2 LRR3 for cellular trafficking (Fig. 3C,D). Both the number of repeats (Fig. 2A-C) and their sequence (Fig. 3A-D) are thus critical for Adgra2 trafficking (Fig. 3C,D). Moreover, a perfect correlation was observed between the capacity of the LRR chimera variants to reach the plasma membrane and their ability to support vascular sprouting in the zebrafish hindbrain or to induce the formation of DRG neurons (Fig. 3E).

### Reck and Adgra2 traffic independently

**Fig. 4.**
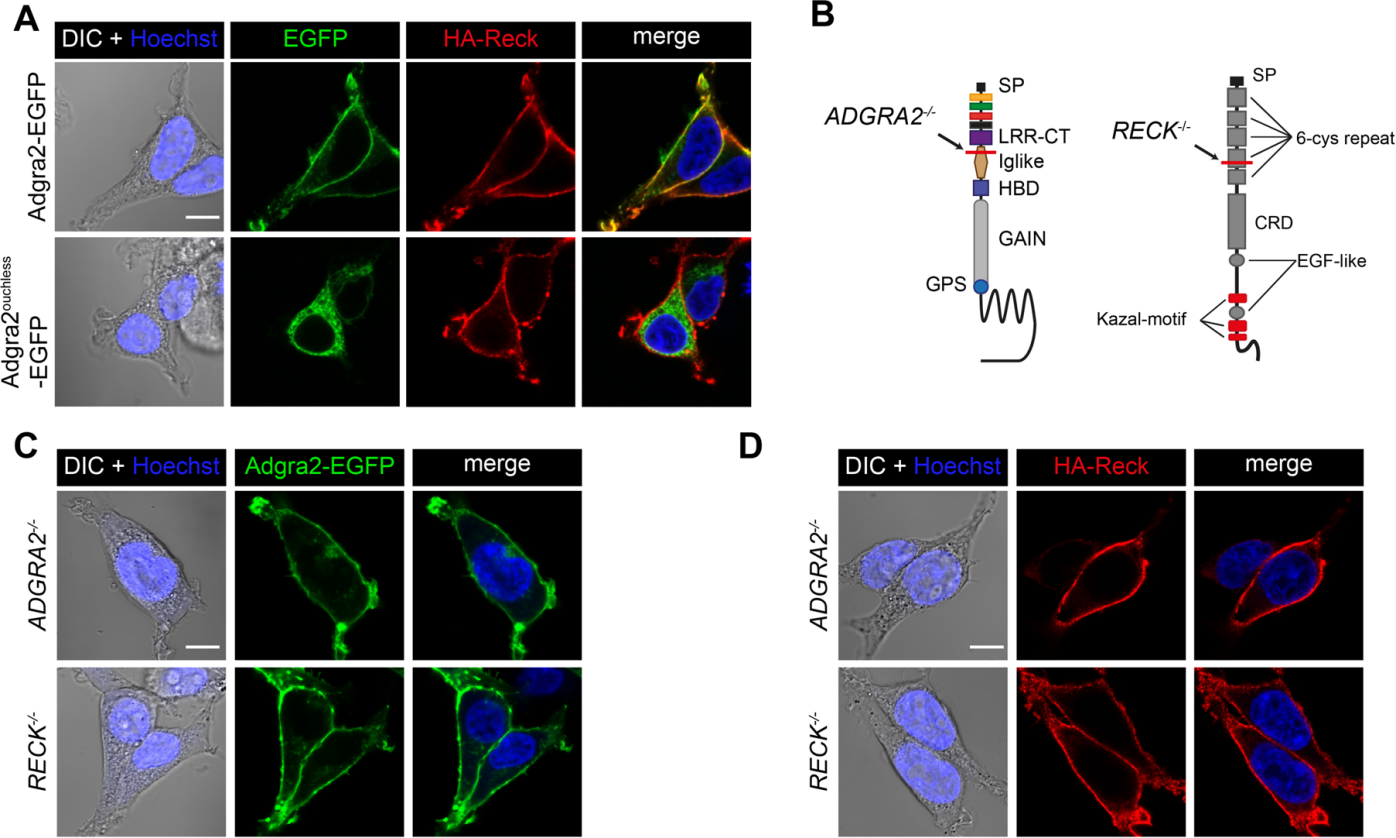
**Independent trafficking of Reck and Adgra2 to the plasma membrane.** (A) Single-plane confocal images of non-permeabilized HEK293T cells 48 h after transfection with *HA-reck* and *adgra2-EGFP* variants, as indicated. (B) Schematic representation of the genetic lesions of *ADGRA2^−/−^* and *RECK^−/−^* cells. The position of the frame-shift mutation is indicated by the red line. See Fig. 1A for schematic labels. (C,D) Single-plane confocal images of non-permeabilized *ADGRA2^−/−^* and *RECK^−/−^* HEK293T cells 48 h after transfection with *adgra2-EGFP* (C) and *HA-reck* (D) constructs. In all panels, EGFP is detected by direct fluorescence and the HA-Reck fusion by anti-HA indirect immunofluorescence. Cells were additionally transfected with a *Wnt7a* (mouse gene) expression construct. Nuclei were counterstained with Hoechst. Scale bars: 10 μm.

When overexpressed in cultured cells, Adgra2 and Reck colocalize at the plasma membrane and proximity ligation assays further suggest that the proteins may directly interact within this compartment to assemble a receptor complex (Vanhollebeke et al., 2015). It remains to be determined whether the partners recognize and assist each other during their progression within the secretory pathway or instead meet at the plasma membrane after independent trafficking events. We took advantage of the ER retention of the LRR/CT variants to address this question. As revealed by indirect immunofluorescence assays in non-permeabilized HEK293T cells, HA-Reck reached the plasma membrane independently of the nature and trafficking status of the co-expressed Adgra2 receptor (Fig. 4A). In addition, when expressed individually in HEK293T cells, Reck and Adgra2 localized to the plasma membrane (Vanhollebeke et al., 2015). These results suggest that Reck does not require Adgra2 in order to reach the plasma membrane and vice versa. However, as HEK293T cells express low levels of endogenous *ADGRA2* and *RECK* (Vanhollebeke et al., 2015; Zhou and Nathans, 2014), this endogenous protein pool might be sufficient to accompany ectopic Reck and/or Adgra2 during secretion. We therefore engineered *ADGRA2^−/−^* and *RECK^−/−^* HEK293T cells through CRISPR/Cas9 approaches and re-evaluated Adgra2 and Reck trafficking in these genetic backgrounds (Fig. 4B, Fig. S1). As in WT cells, both proteins accumulated at the plasma membrane when expressed individually, indicating that each partner can reach its final destination independently (Fig. 4C,D).

**Fig. 5.**
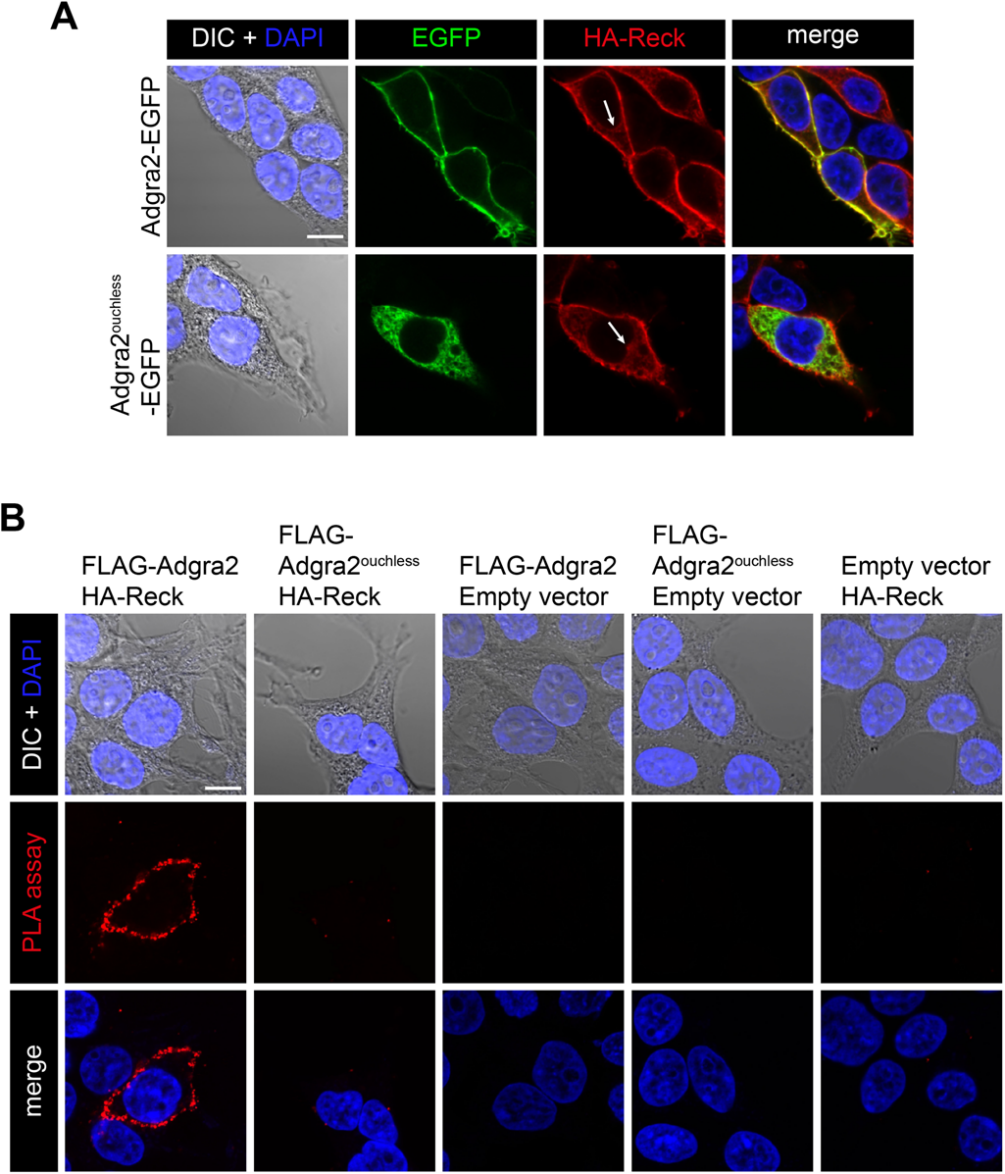
**Cellular distribution of Adgra2 and Reck interaction.** (A) Single-plane confocal images of saponin-permeabilized HEK293T cells 48 h after transfection with *HA-reck* and *adgra2-EGFP* variants, as indicated. Nuclei were counterstained with DAPI. EGFP is detected by direct fluorescence and the HA-Reck fusion by anti-HA indirect immunofluorescence. Arrows point to the ER. (B) Proximity ligation assays in HEK293T cells 48 h after transfection with *FLAG-adgra2*, *FLAG-adgra2^ouchless^* and *HA-reck* constructs, as indicated. Nuclei were counterstained with DAPI. In all panels, cells were additionally transfected with a *Wnt7a* (mouse gene) expression construct. Scale bar: 10 μm.

When assessed 48 h post transfection in saponin-permeabilized HEK293T cells, a minor fraction of HA-Reck can be immunodetected in the ER and as such co-distributes with Adgra2^ouchless^ (Fig. 5A, arrows) and presumably with a fraction of WT Adgra2 transiting through this compartment. To test whether Adgra2 is able to interact with Reck under these conditions, we performed proximity ligation assays as described previously (Vanhollebeke et al., 2015). As shown in Fig. 5B, no interaction could be detected between HA-Reck and FLAG-Adgra2^ouchless^, in contrast to the plasma membrane-localized signal readily detected in HA-Reck and FLAG-Adgra2 co-expressing cells. These results suggest that either the ER is not permissive for the formation of the complex or that the LRR deletion in Adgra2 impairs its interaction with Reck.

## DISCUSSION

Adgra2 and Reck are recently recognized synergistic activators of Wnt7-stimulated Wnt/β-catenin signaling acting in CNS-invading ECs and neural crest-derived cells of the zebrafish DRG. They have been proposed to contribute to the assembly of a Wnt7 receptor complex at the plasma membrane operating either as a stand-alone receptor complex or in association with the classical Fzd/Lrp5/6 receptors (Noda et al., 2016; Vanhollebeke et al., 2015), but very limited information is thus far available on their mechanism(s) of action. We therefore need to better understand the cellular and molecular modalities of Adgra2/Reck interaction and determine whether the partners cooperate beyond their suspected role as co-receptors at the plasma membrane.

To this end, we characterized here the functionally null mutation of *adgra2* recently identified in zebrafish *ouchless* mutants. The genetic lesion results in *adgra2* alternative splicing and we show in this work that this receptor variant localizes to the ER instead of the plasma membrane. This unprecedented occurrence of an aberrantly routed Adgra2 prompted us to evaluate whether the intracellular trafficking of Reck and Adgra2 are interdependent. When co-expressed with the ER-retained Adgra2 variant, Reck still reached the plasma membrane. Extending this analysis in CRISPR/Cas9 engineered cells, Reck was shown to accumulate at the plasma membrane in both WT and *ADGRA2*^−/−^ HEK293T cells and, similarly, Adgra2 trafficking to the plasma membrane was unaffected by the presence or absence of RECK. These data indicate that the partners, when expressed individually, are able to traffic independently. When co-expressed in HEK293T cells, their close proximity can be detected by PLA assays at the plasma membrane but not within the endomembrane compartments of the secretory pathway through which they transit. These combined observations indicate that the partners first meet at the plasma membrane and that their synergy is likely restricted to the events occurring subsequently at the cell surface, in agreement with the current model (Noda et al., 2016; Vanhollebeke et al., 2015). It is conceivable that the interaction between Adgra2 and Reck is only made possible within plasma membrane microdomains of specific proteolipidic composition or that a yet to be defined component induces complex formation within this compartment. The selective association of Reck and Adgra2 at the plasma membrane could also result from the higher concentrations reached within this final membrane compartment favoring the potentially transient encounters of the partners.

The ER retention of the *ouchless* variant of Adgra2 results from an aberrant splicing event leading to in-frame deletion of the LRR3 motif. Through the interrogation of a collection of LRR deletion and chimeric variants, this work reveals that both the number and primary sequence of the four tandemly-arranged LRR motifs composing the LRR domain are important for Adgra2 trafficking. Functional characterization of chimeras in which LRR3 is replaced by LRR units from the same structural subfamily suggests that specific residues within LRR3 have essential functional roles. Chimeras harboring the closely related Adgra3 LRR3 (71% similarity) maintain functionality while chimeras with the more distantly related Adgra2 LRR1, LRR2 (54% similarity) and CPN2 LRR7 (42% similarity) do not. No other N-terminal domain of Adgra2 appears to be required for Adgra2 localization at the plasma membrane, underlying the specific requirement for the LRR/CT domain in this process.

The LRR/CT-dependent Adgra2 localization results are in apparent conflict with previous findings. Indeed, Posokhova et al. analyzed the subcellular distribution of a set of N-terminal deletion variants of ADGRA2 and reported a robust trafficking mechanism to the plasma membrane independent of any of the extracellular domains, including the LRR/CT domain (Posokhova et al., 2015). We note however that the smallest N-terminal truncation variant analyzed by Posokhova et al. (labeled ΔLRR) encompassed the LRR C-terminal domain and hence corresponds to the ΔLRR/CT nomenclature used in this work. As this variant exhibits intermediate phenotypes under the experimental conditions of this study, with most proteins localizing within the ER and a subfraction at the plasma membrane, it is conceivable that its enrichment in the ER remained unnoticed in the absence of quantitative colocalization analysis. Importantly, the critical role of the LRR/CT domain and subdomains thereof on Adgra2 trafficking was confirmed in all tested cellular settings, both *in vivo* (blastomeres, ECs) and *in vitro* (HEK293T cells).

Mechanistically, the ER retention of Adgra2 molecules exhibiting LRR variations or deletions is most easily explained by improper folding. As (i) receptor variants lacking the entire LRR/CT domain accumulate in the ER and (ii) the deletion of the adjacent Ig-like domain is sufficient to suppress the defective trafficking of LRR/CT variants, we propose that improper folding of the Ig-like domain and not the LRR/CT domain itself is causing ER retention of the LRR/CT variants. The LRR/CT domain might help instruct folding of the adjacent Ig-like domain through intramolecular contacts involving specific residues within the LRR domain.

While this study describes the role of the LRR/CT domain in promoting Adgra2 progression through the ER, it does not exclude additional roles for this domain in the Adgra2/Reck signaling pathway. The LRR/CT domain might, for instance, be additionally implicated in the interactions with Reck, Wnt7 or Fzd/Lrp5/6 occurring at the plasma membrane. By extension, the contribution of the other extracellular domains of Adgra2 and Reck to Adgra2/Reck signaling will require further investigation. These future studies will be important not only for their insights into the molecular mechanisms governing essential developmental processes, but also because they hold the key to understanding the thus-far elusive mechanisms of Wnt ligand-specific signaling pathways.

## MATERIALS AND METHODS

### Zebrafish strains and cell lines

Zebrafish (*Danio rerio*) were raised and maintained under standard conditions. The following lines were used: *Tg(kdrl:GFP)^s843^* (Jin et al., 2005), *Tg(kdrl:ras-mCherry)^s896^* (Chi et al., 2008), *Tg(fliep:Gal4FF)^ubs4^* (Herwig et al., 2011), *Tg(-17.0neurog1:EGFP)^w61^* (McGraw et al., 2008) and *adgra2^s984^* (Vanhollebeke et al., 2015). All animal experiments were performed in accordance with the rules of the State of Belgium (protocol approval number: CEBEA-IBMM-2012:65). HEK293T cells were obtained from ATCC (CRL-3216) and were not further authenticated or tested for contamination.

### Cloning strategy, morpholino and RNA expression constructs

The *adgra2* deletion mutants and hybrids were generated by In-Fusion cloning (Takara, Mountain View, CA). Deletions correspond to the following amino acids: Adgra2^ΔLRR1^: 77-100; Adgra2^ΔLRR2^: 101-124; Adgra2^ΔLRR3^: 125-148; Adgra2^ΔLRR4^: 149-169; Adgra2^ΔLRR^: 70-166; Adgra2^ΔLRR/CT^: 70-228; Adgra2^ΔIg-like^: 230-344; Adgra2^ΔHRM^: 353-406; Adgra2^ΔGAIN^: 416-727; Adgra2^ΔLRR/CT/Ig-like^: 70-344; Adgra2^ΔLRR/CT/Ig-like/HRM^: 70-406. Chimeras were generated by substituting the Adgra2 LRR3 (125-148) with Adgra3 LRR3 (122-145), CPN2 LRR7 (247-270), Adgra2 LRR1 (77-100) or Adgra2 LRR2 (101-124). Capped messenger RNA was synthesized using the mMESSAGE mMACHINE kit (Ambion, Carlsbad, CA). One-cell stage embryos were injected either with 100 pg of the indicated mRNA or 4 ng of a previously validated *adgra2* splice-blocking morpholino (Vanhollebeke et al., 2015). The EGFP C-terminal Adgra2 fusions were generated by directly linking the coding sequences of EGFP to the last amino acid-coding codon of *adgra2* in pCS2 by In-Fusion cloning (Takara). The fusion products were then subcloned in a pTol2-5xUAS expression construct.

### Transgenic endothelial mosaic expression

Transgenic endothelial mosaic overexpression was achieved by co-injecting 25 pg of Tol2 transposase mRNA and 25 pg of the pTol2-*5xUAS:adgra2-EGFP*, pTol2-*5xUAS:adgra2^ouchless^-EGFP* and pTol2-*5xUAS:adgra2^ΔLRR3^-EGFP* constructs into double transgenic *Tg(kdrl:ras-mCherry)^s896^*; *Tg(fliep:Gal4FF)^ubs4^* embryos at the one-cell stage.

### Cell transfection, immunofluorescence and proximity ligation assay (PLA)

The following antibodies were used: mouse monoclonal anti-FLAG M2, 1:10,000 for PLA (F1804, Sigma-Aldrich, St. Louis, MO), purified polyclonal rabbit anti-HA, 1:400 for PLA and 1:250 for immunofluorescence assays (H6908, St. Louis, MO) and anti-rabbit Alexa594-conjugated secondary antibody, 1:5000 (Molecular Probes, Carlsbad, CA). DAPI and Hoechst counterstaining were performed for 2 min at 5 µg ml^−1^ and 10 µg ml^−1^, respectively. HEK293T cells were transfected with Lipofectamine 2000 (Life Technologies, Carlsbad, CA) and grown in glass-coated imaging chambers (Ibidi, Martinsried, Germany) for 48 h before being fixed for 15 min in 4% paraformaldehyde at room temperature. Where indicated, cells were permeabilized with 0.5% saponin in PBS for 10 min at room temperature. PLA assays were performed following the manufacturer's instructions (Sigma-Aldrich, St. Louis, MO).

### Generation of *ADGRA2^−/−^* and *RECK^−/−^* HEK293T clones

CRISPR/Cas9 guide sequences were cloned in pSpCas9(BB)-2A-GFP (Ran et al., 2013). GFP^+^ HEK293T cells were isolated 48 h after transfection by FACS (AriaIII, BD Biosciences) and clonally expanded before homozygous mutant selection by high resolution melt analysis and lesion identification through Sanger sequencing.

### Imaging

Images were acquired on a Zeiss LSM710 confocal microscope. Colocalization was assessed using the Pearson correlation coefficient (PCC) calculated with coloc2 in ImageJ (NIH). Applied to colocalization analysis, PCC is a measure of the linear correlation between the intensities observed in the two channels of interest. A value of 1 indicates that the two signals are positively and perfectly linearly related; a value of −1 indicates a perfectly linear, but negative correlation; and a value close to zero indicates that the distributions of the two signals are uncorrelated.

### Statistical analysis

Statistical analyses were performed using the GraphPad Prism software. Sample size was determined with the G*power 3.1.5 software to reach adequate statistical power. Each dot plot value represents an independent cell or embryo and every experiment was conducted three times independently. *P*-values were calculated by the Kruskal–Wallis test (post hoc Dunn's test).
